# *In vivo* evidence of outer hair cell length changes and their role in high-frequency cochlear mechanics

**DOI:** 10.3389/fauot.2025.1617134

**Published:** 2025-09-08

**Authors:** Sunil Puria, Nam Hyun Cho, John J. Guinan

**Affiliations:** 1 Eaton Peabody Laboratories, Massachusetts Eye and Ear, Boston, MA, United States,; 2 Department of Otolaryngology-HNS, Harvard Medical School, Boston, MA, United States,; 3 Speech and Hearing and Bioscience and Technology Graduate Program at Harvard University, Cambridge, MA, United States

**Keywords:** inner ear, cochlear mechanics, outer hair cell (OHC), optical-coherence-tomography, cochlear amplification

## Abstract

**Introduction:**

The great sensitivity and frequency selectivity of mammalian hearing originates in cochlear mechanics. Cochlear responses to sound are thought to be amplified by outer hair cells (OHCs) cyclically expanding and contracting lengthwise in response to audio-frequency changes in their transmembrane potential. The mechanism by which OHCs amplify sound in the cochlea remains an enigma. Individual OHCs in micro-chambers have low-pass current-to-displacement characteristics with corner frequencies of a few kHz. ***In vivo***, OHC corner frequencies were estimated to be ~3 kHz. Since cochlear motion is amplified at many tens of kHz, the low OHC corner frequency appeared to be a problem for high-frequency amplification to be produced by OHC cyclic length changes.

**Methods:**

We compared OHC motion to BM motion using high-resolution optical-coherence-tomography (OCT) in the 38–46 kHz best-frequency (BF) region of Mongolian gerbils. We measured transverse organ-of-Corti (OoC) motions at the OHC top near the reticular lamina (RL), at the OHC bottom near the OHC-Deiters-cell junction (ODJ), and at the basilar-membrane (BM). From these *in-vivo* measurements we determined the transverse (lengthwise) motions of individual OHCs.

**Results:**

At moderate tone levels and at frequencies up to 40–50 kHz, the motions at the top and bottom of the OHCs were almost exactly out of phase, consistent with motion generated by OHC motility. Furthermore, OHCs length changes were greater than BM motion at frequencies of 40–50 kHz.

**Discussion:**

Thus, a low OHC current-to-displacement corner frequency should not be viewed as preventing OHCs from adding energy cycle-by-cycle to overcome cochlear damping and provide cochlear amplification. Our results are consistent with cochlear amplification being produced by OHC motility at all frequencies.

## 1 Introduction

It is well-established that cochlear outer-hair cells (OHC) have piezo-electric like electromotile properties (Brownell et al., 1985; Mountain and Hubbard, 1994; Gale and Ashmore, 1994). Electrical stimulation of OHCs can produce forces and motion up to 80 kHz (Frank et al., 1999), which is consistent with the hypothesis that OHC electromotility pumps power cycle-by-cycle into the cochlea thereby amplifying responses over the wide frequency range of mammalian hearing (Robles and Ruggero, 2001; Guinan et al., 2012; Wang et al., 2016). However, experiments on isolated OHCs in micro-chamber environments directly measured OHC electro-motility and found that the conversion of OHC stereocilia current to OHC transmembrane voltage had ms-long time constants (Santos-Sacchi, 1989; Housley and Ashmore, 1992). These long time constants were attributed to the OHC basolateral membrane having a low-pass RC-filter corner frequency (RC: resistance times capacitance) which seemed too low to allow OHCs to add eneregy cycle-by-cycle at high frequencies; this was called the OHC time-constant “problem” (Santos-Sacchi, 1989). In more recent experiments done with experimental refinements, voltage-controlled, OHC sound-frequency motion was found up to ~20 kHz (Santos-Sacchi, 1992; Santos-Sacchi and Tan, 2018, 2019; Santos-Sacchi et al., 2023), but this is still much lower than the frequencies at which there is cochlear amplification, i.e., 40–50 kHz in gerbils (Cho and Puria, 2022).

OHCs must have appropriate loading for efficient energy transfer at high frequencies (Iwasa, 2017; Rabbitt, 2020). Micro-chamber measurements lack the load of *in vivo* OHCs. OHC-load coupling can change the effective OHC corner frequency (Mountain and Hubbard, 1994; Iwasa, 2017). However, *in vivo* measurements at 13–25 kHz best frequency (BF) locations, of distortion products in gerbils indicated a low-pass OHC corner frequency of ~3 kHz (Vavakou et al., 2019; van der Heijden and Vavakou, 2021). This *in vivo* 3 kHz corner frequency is higher than micro-chamber measurements but still below the tens of kHz at which small mammals can hear. *In-vivo* measurements of locally-generated harmonic distortion in mice at 9 kHz showed out-of-phase OHC top-to-bottom differential motion extending to 20 kHz, with amplitude decreasing above 9 kHz (Dewey et al., 2021). No direct, *in vivo* measurements of sound-evoked OHC top-to-bottom motion from a location with a BF of several tens of kHz have been made, nor have there been direct comparisons of OHC motion magnitude to BM motion at a high-BF location and at a frequency with cochlear amplification.

As part of our continuing study of organ-of-Corti (OoC) motion in the gerbil base (Cho et al., 2022; Cho and Puria, 2022), we measured motions at the 38–46 kHz BF region with a viewing angle that is more transverse than previous measurements (e.g., Cooper et al., 2018). Our approach allowed us to measure transverse motions at both ends of the OHC axial length, i.e., at the apical end, near the reticular lamina (RL), and at the basal end, near the OHC-Deiter-cell (DC) junction (ODJ). Our optical-coherence-tomography (OCT) system allowed measurements, in the same animal, across individual OHCs in each of the three OHC rows with a relatively high axial resolution of ~2.23 μm (Cho and Puria, 2022). We also measured basilar-membrane (BM) motion, so that the OHC top-to-bottom length changes can be compared to BM motion at the same frequency and location. These measurements allowed us to show that OHCs change length in response to tones at frequencies up to 40–50 kHz, with length-change amplitudes that were larger than BM motion. A preliminary report of this work is Cho and Puria (2022).

## 2 Materials and methods

### 2.1 Experimental design and statistics

We measured motions at the top and bottom of individual OHCs and calculated the OHC top-to-bottom differential motion: ΔOHC = RL – ODJ. In a normal cochlea, OHC electromotility is excited by cyclic BM motion that tilts the OoC and deflects OHC stereocilia, which results in current flow into the OHC, a OHC transmembrane voltage change, and cyclic OHC length changes. Since the BM motion is produced by the traveling wave, the OHC motion, ΔOHC, has the phase delay of the traveling wave, which is several cycles at frequencies near BF. Thus, measuring ΔOHC gives the amplitude of the OHC top-to-bottom motion with the phase of the traveling wave. ΔOHC does not provide a good metric of the OHC top-to-bottom phase *difference*. A better metric for this is *θ*_OHC_, which is the phase difference: RL phase minus ODJ phase.

If OHC motion were primarily produced by following BM transverse motion without a contribution from OHC electromotility (i.e., OHC length was constant), then the motions at the top and bottom of an OHC would be in phase, with both motions approximately proportional to the BM motion. In contrast, if OHC motion is primarily produced by OHC electromotility (i.e., OHC length changes), then the motions at the top and bottom of an OHC would be close to out of phase (i.e., differ by 0.5 period) as internal OHC forces push the RL side up and the ODJ side down. Thus, we examine the *θ*_OHC_ phase (RL phase minus ODJ phase) for evidence of OHC electromotility (phase difference about 0.5 period) vs. whether OHC motion primarily came from following BM motion (phase difference 0.0 period).

Although the ΔOHC motion phase is dominated by the traveling-wave phase, its magnitude can still be compared to the magnitude of the BM motion. If the OHC RC filter has rendered OHC motility to be unimportant at frequencies near BF (38–45 kHz in our experiments), we would expect that ΔOHC magnitude would be much less than the BM-motion magnitude. To examine this issue, we also calculated ΔOHC/BM, which is (RL-ODJ)/BM.

At frequencies near BF, calculations of (RL-ODJ)/BM were limited by the ODJ motion at low sound levels being low in amplitude and not passing our signal-to-noise ratio (SNR) criterion (downward ODJ motion was sometimes accompanied by upward BM motion, which made the overall ODJ motion small so that it didn’t pass the SNR criterion). In the 40–50 kHz range (most of which was above the local BF), accurate data were available in different animals at different frequencies within this range because the points with low SNRs occurred at different frequencies in different animals. In addition, the data are not expected to have a normal distribution. Because of these issues, standard statistical tests (e.g., *t*-test) are not applicable.

For the present data set, one kind of statistical test that can be done is a permutation test, also known as the bootstrap, rerandomization test or shuffle test (e.g., Efron and Tibshirani, 1993). A permutation test at each sound level was done using the *N* points for which (RL-ODJ)/BM could be calculated across multiple animals. At each level we did 1,000,000 trials in which a (RL-ODJ)/BM pseudo-average was obtained by averaging sets of *N* points chosen randomly, with replacement, from the *N* data points. The trial was scored as a “failure” if the (RL-ODJ)/BM pseudo-average value was below the criterion value. The probability that the average was less than the criterion value was the number of failures divided by the number of trials.

Below we state the methods essential for understanding our results. Our methods were described by Cho et al. (2022) and Cho and Puria (2022) and additional details can be found there.

### 2.2 OCT imaging and vibrometry

OCT measurements were conducted using a commercial system (GAN620C1, Thorlabs, Germany). This system combined two near-infrared superluminescent-diode light sources with center wavelengths of 847.5 and 929.6 nm. The spectral bandwidths of these sources were 63.4 and 95.8 nm, respectively.

**FIGURE 1:**
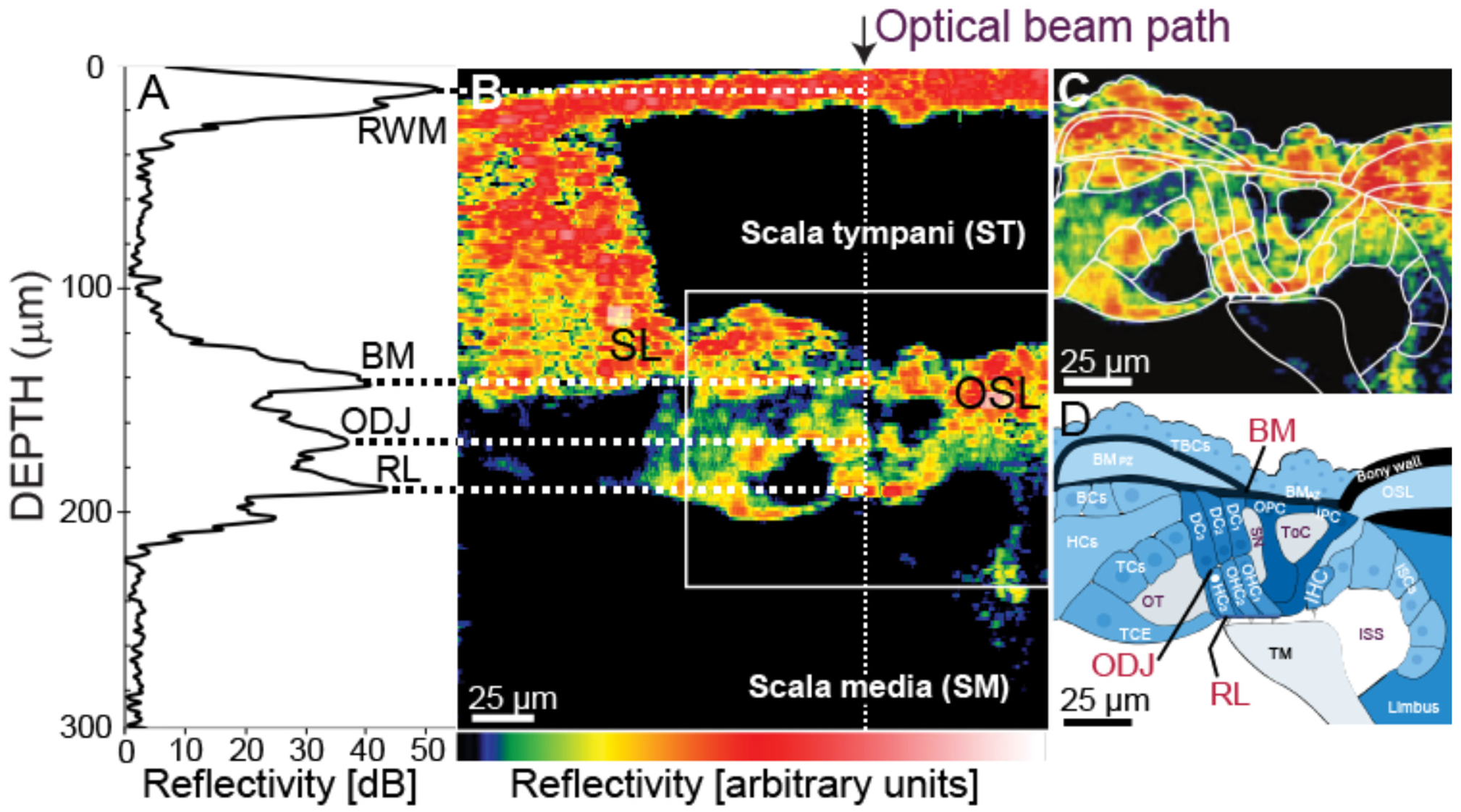
The relationship of the optical-coherent-tomography (OCT) beam to cochlear anatomy. **(A)** An OCT backscattered-light depth profile from a 1D “A-scan” [at the white dotted line in **(B)**]. The peaks correspond to the round-window membrane (RWM), the basilar-membrane (BM) at the arcuate-pectinate junction, the outer hair cell (OHC) Deiters cell (DC) junction (ODJ), and the reticular lamina (RL). **(B)** A through-the-RWM, ***in vivo***, cross-sectional, OCT B-scan image from gerbil G614. Color = reflectivity strength (arbitrary units, key at bottom, white is highest). **(C)** Enlarged view of white box in **(B)** with organ of Corti (OoC) structures outlined. The outlined TM was identified by increasing the image-to-color-map gain (not shown). **(D)** Outlined structures labeled. TBCs, tympanic border cells; BCs, Boettcher cells; OSL, osseous spiral lamina; HCs, Hensen’s cells; TCs, tectal cells; TCE, tectal-cell extension; ISCs, inner-sulcus cells; IHC, inner hair cell; TM, tectorial membrane; OT, outer tunnel; SN, Space of Nuel; ToC, tunnel of Corti; ISS, inner spiral sulcus.

Using a 0.055-NA objective (OCT-LK3-BB, Thorlabs, Germany), this system yielded an axial resolution of 2.23 μm (in water) and a lateral resolution of ~8 μm. Custom LabVIEW (NI, TX, USA) software (VibOCT version 2.1.4) controlled the OCT data acquisition at a frame rate of 192 kHz and FFT Length of 8,192 corresponding to 42.7 ms acquisition time. The system provided video images of the tissue that guided the anatomical approach (Figure 1B). A single “A-scan” measured reflected light vs. depth (e.g. Figure 1A). “M-scans” for vibrometry were a series of A-scans done multiple times with their phases synchronized to the tone. A Fast Fourier Transforms (FFT) on each A-scan of the M-scan yielded a reflectivity depth profile (e.g., Figure 1A). A second FFT along the time axis of the multiple depth profiles, at one depth of the interferometric data, extracted the vibration displacement and phase at that OoC location. OCT accuracy was evaluated by comparing the OCT-system measurements with measurements from a calibrated laser-Doppler vibrometer (LDV; Polytec OFV501/OFV2600, Irvine, CA) using motion from a piezoelectric vibrator. The OCT and LDV measurements were typically within 1 dB (~2 dB at some frequencies) and had a systematic phase differences that was compensated. In a typical experiment, after the measurements at a particular place were done, the noise floor was assessed by measuring the motion at that place with no sound applied. Noise floors ranged from 100 to 500 pm; more reflective structures had lower noise floors. Measurements were only used if the signal-to-noise ratio (SNR) was 6 dB, or more.

Overall, our OCT system provided better spatial resolution than the resolution obtained in previously published OCT OoC measurements (e.g, Figure 1C), primarily because our OCT system had a shorter infrared wavelength and a wider bandwidth. In contrast to some other OoC systems, we obtained a refection peak at the position of the RL (possibly because of our shorter OCT wavelength and/or that our more-transverse viewing angle made the RL reflect light better). Our system was able to resolve the tops and bottoms of each of the three OHC rows (Cho and Puria, 2022). However, we have the most data for OHC row 3, so we use OHC-row-3 data for comparisons across animals. For the point being made here, only data from one OHC row is necessary. Most OoC images reported in the literature are from postmortem tissue. Figure 1D shows the OoC schematic obtained from the in vivo image shown in Figure 1C.

### 2.3 Animal preparation

We used female Mongolian gerbils (*N* = 19, aged 5–11 weeks, weight range 41–76 g). Our results are from seven left ears with good cochlear sensitivity as assessed by distortion-product otoacoustic emissions (DPOAEs; these data are shown in Cho and Puria, 2022, Supplementary material). Anesthesia was induced by intraperitoneal injection of sodium pentobarbital (70 mg/kg), followed by a subcutaneous injection of acepromazine (1 mg/kg) mixed with atropine (0.06 mg/kg). The depth of anesthesia was evaluated every 30–60 min via toe-pinch response and/or an increase in heart rate of >10%. To maintain anesthesia, 1/3 the initial dose of sodium pentobarbital was given every 45–60 min. This study was approved by the MEE Institutional Animal Care and Use Committee (IACUC), PHS Approved Animal Welfare Assurance D16–00215 (A3332–01). All methods and procedures were performed in accordance with the approved MEE protocol and written up following the Animal Research: Reporting of the *In Vivo* Experiments (ARRIVE) guidelines.

Anesthetized gerbils were fixed by a headholder and placed on a heating pad that maintained rectal temperature at 38.5 ± 1 °C. The left pinna and external ear canal were removed. The tympanic bulla was exposed and opened using a ventral approach. The tympanic membrane, malleus, incus, stapes, and round-window membrane (RWM) were kept intact.

After surgery, the animal was placed on a two-stage goniometer (07-GON-503, Melles Griot, Carlsbad, CA, USA) positioned on top of a three-axis micro-manipulator (OCT-XYR1, Thorlabs, Germany) mounted on a vibration-isolation table. The head was oriented to make measurements through the intact RWM with the viewing angle adjusted to be close to transverse to the BM. This angle provided access to BFs in the 38–46 kHz range. The BM motions used in this paper were from the arcuate-pectinate junction, which yielded the largest BM motions.

After *in vivo* measurements, animals were euthanized by intraperitoneal injection of Fatal Plus (>150 mg/kg). Within 5–10 min after the injection, the animal stopped breathing and the heart typically stopped. Postmortem measurements were done 5–60 min after the animal stopped breathing and had no heartbeat.

### 2.4 Stimulus generation and acoustic measurements

Signal generation and measurement used a National Instruments (NI) PXI-4461 in an NI PXI-1031 chassis with an NI PXI-8196 computer (NI, TX, USA) at a sample rate of 192 kHz with an FFT length of 8,192. OCT vibrometry used a sequence of pure tones (2–63 kHz, ~0.8-kHz linear frequency steps) generated by custom software (SyncAv, version 0.42). The board output was amplified by a Techron Model 5507 amplifier (AE Techron, IN, USA) that drove a Parts Express 275–010 tweeter in a closed-field acoustic assembly. Ear-canal pressure was measured by a calibrated Knowles FG-23329 microphone coupled to a probe tube that extended to 1–2 mm from the tympanic membrane.

At the start of the experiment, we measured the *in situ* ear-canal sound pressure as a function of frequency from a constant stimulus voltage. The measured pressure was then used to set the stimulus voltage to produce a nearly flat sound level across frequency. This equalization voltage was scaled in ~10 dB steps (or ~5 dB steps above 85 dB SPL) to produce the tone levels used. The nominal sound pressure level in SPL (0 dB SPL = 20 μPa) associated with each measurement was calculated as the average SPL measured across frequency for a given set of equalization voltages. The equalization voltages were varied to set the nominal SPLs from ~30 to ~90 dB across experiments, while the actual SPLs typically varied ±4 dB due to small differences across time in the position of the acoustic assembly or fluid in the ear canal.

One consequence of making measurements at high frequencies (>30 kHz) is that small differences in the acoustics can make substantial differences in the ear-canal sound pressure, especially near acoustic resonances. Changes across time in sound-pressure for the same stimulus voltage were minimized by measuring the sound-pressure stimulus and OCT vibrations simultaneously, and by expressing vibrometry measurements as *gains* (i.e., OoC-motion displacement/SPL). Measurements at different structures, which were done at separate times, were then compared using their gains. Full complex-number computations were used (e.g., computations were done using real and imaginary parts, not computations using amplitude and phase).

## 3 Results

**FIGURE 2:**
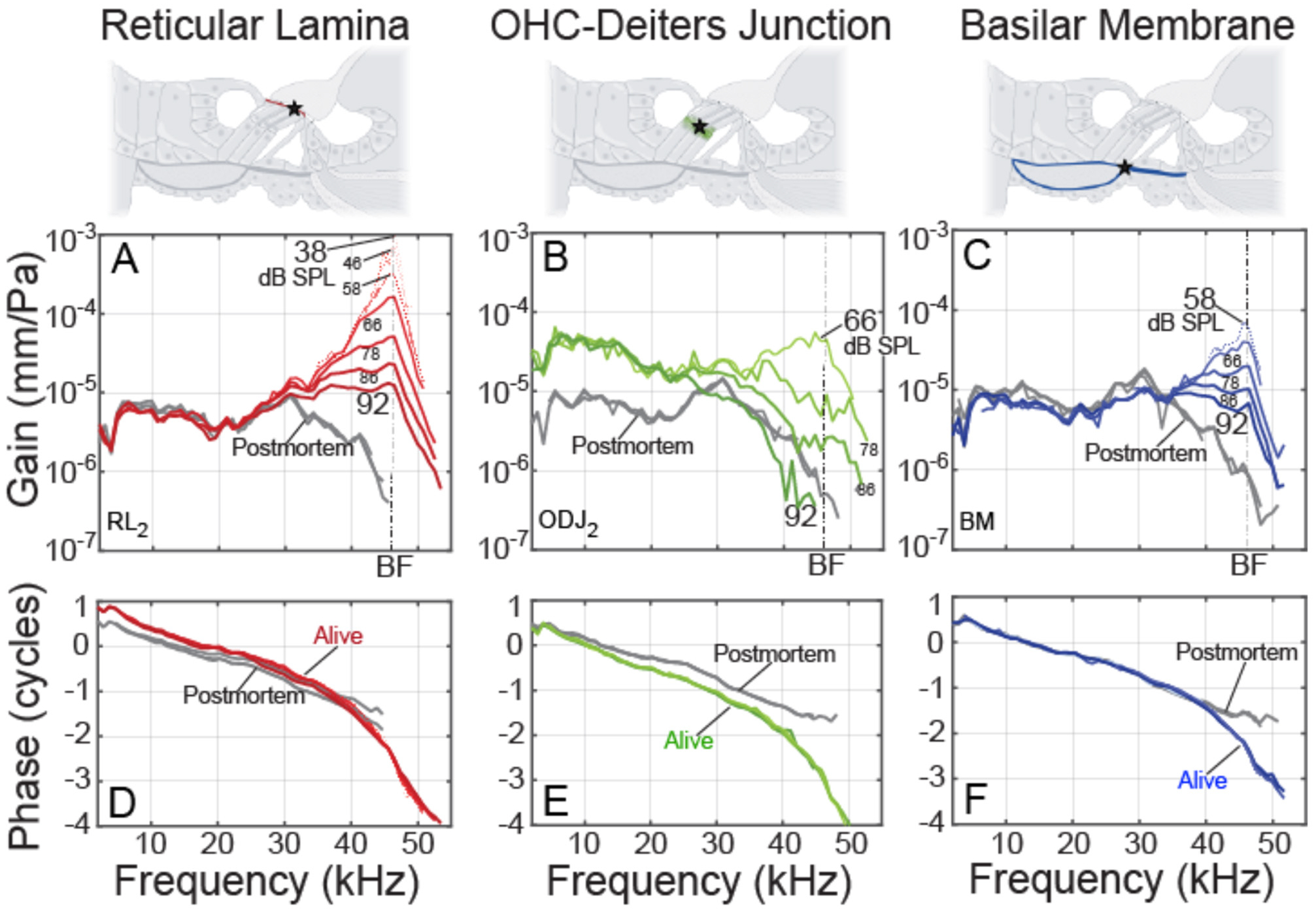
Example cochlear motion gains from gerbil 637 at various sound levels (thinner lines = lower levels), as functions of tone frequency. Gain = displacement in mm divided by ear-canal sound pressure in Pascals. Columns: **(A, D)** reticular lamina (RL), **(B, E)** outer-hair-cell Deiters-cell junction (ODJ) and **(C, F)**, basilar membrane (BM) at the arcuate-pectinate junction. Top Row: cochlear cross-section diagrams with stars showing the measurement locations. Middle Row: gain magnitudes. Bottom Row: gain phases. Note that the RL, ODJ and BM postmortem phase data overlap when plotted on the same graph. RL and ODJ from second row OHCs. BF, best frequency.

Example data at various sound levels are shown in Figure 2. As explained in Section 2, the motion gains (displacement/sound pressure) provide the most accurate metrics across structures, so motion differences were characterized using gains, not displacements or velocities. Figure 2 shows that at frequencies near the BF, all three regions had gains with compressive nonlinearities. At low sound levels, the gains were at least 30 dB more than the postmortem gain. In contrast, at frequencies an octave or more below BF, the gains varied little over the levels tested, and the RL and BM gain magnitudes were close to their postmortem values, while the ODJ had gain amplitudes ~15 dB above its postmortem value. This pattern was representative of our data (Cho and Puria, 2022) and can be understood using the transverse-organ-of-Corti (TOoC) Model (Guinan et al., 2025). All motions showed the classic traveling wave phase accumulation of 2–3 cycles at the BF (Figure 2, bottom), as was found in previous reports (Ren et al., 2016; Dong and Olson, 2013; Cooper et al., 2018).

### 3.1 Outer-hair-cell motion

**FIGURE 3:**
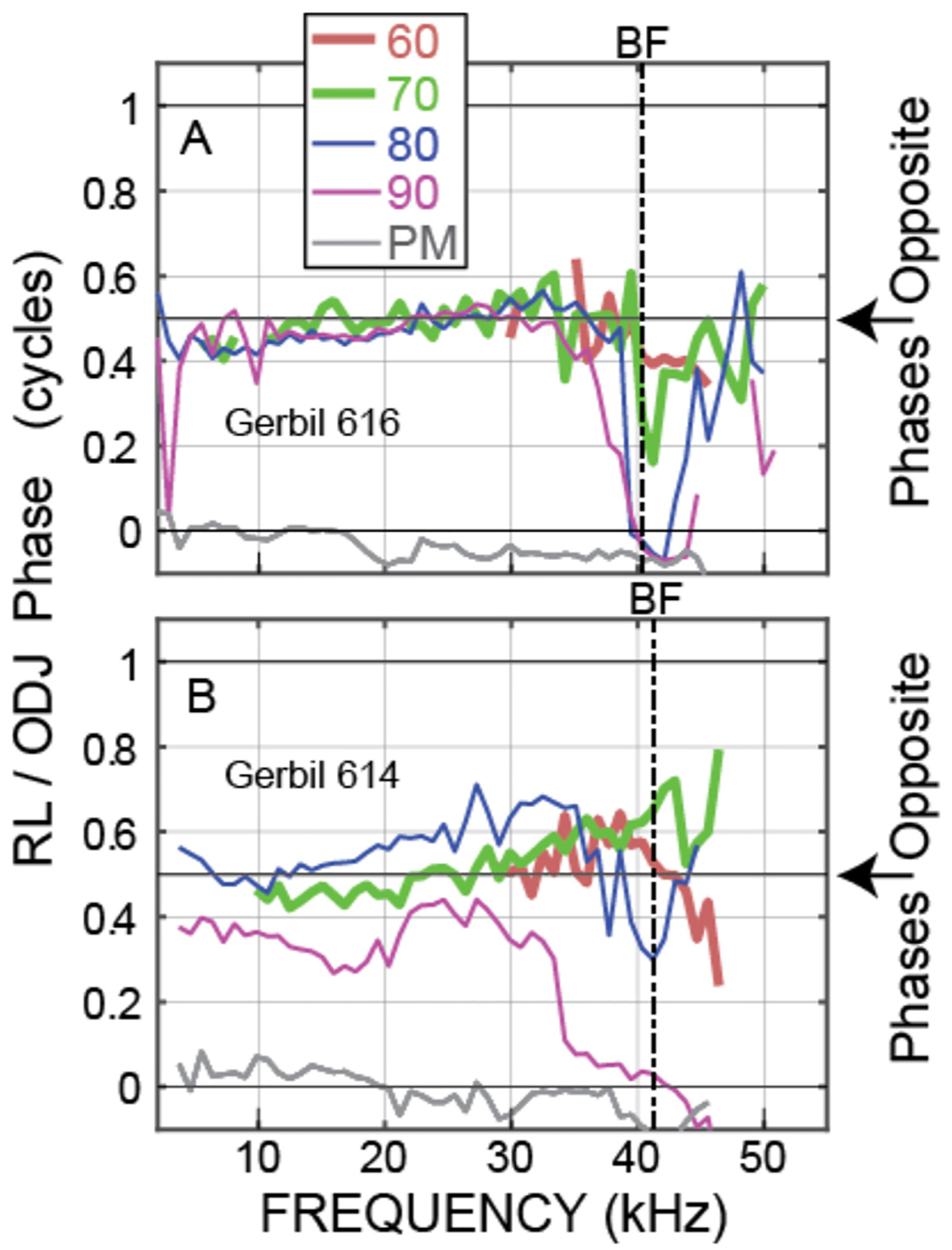
*θ*_OHC_ phase, i.e., the phase of the motion at the OHC ***top*** (the RL) relative to the motion at the OHC ***bottom*** (the ODJ), at various sound levels (key at top shows nominal sound level in dB SPL, PM = postmortem). Data from OHC row 3 of **(A)** Gerbil 616. **(B)** Gerbil 614.

Our main interest is the OHC motion, both the phase difference between the OHC tops and bottoms and how the magnitude of OHC motion compares to the magnitude of BM motion. The best metric for showing the OHC top-to-bottom phase difference is *θ*_OHC_ = PHASE{RL/ear-canal-pressure} minus PHASE{ODJ/ear-canal-pressure}, which cancels out both the ear-canal sound pressure and the traveling-wave phases. Example *θ*_OHC_ phases from two animals are shown in Figure 3. The motions at the tops and bottoms of the OHCs were almost opposite in phase from low frequencies up to near BF (at BF the large transverse traveling-wave motion sometimes dominated the ODJ motion), and above BF at frequencies up to near 50 kHz (Figure 3). Note that in the postmortem animals, the OHC top-to-bottom phase differences were near zero (gray lines in Figure 3).

**FIGURE 4:**
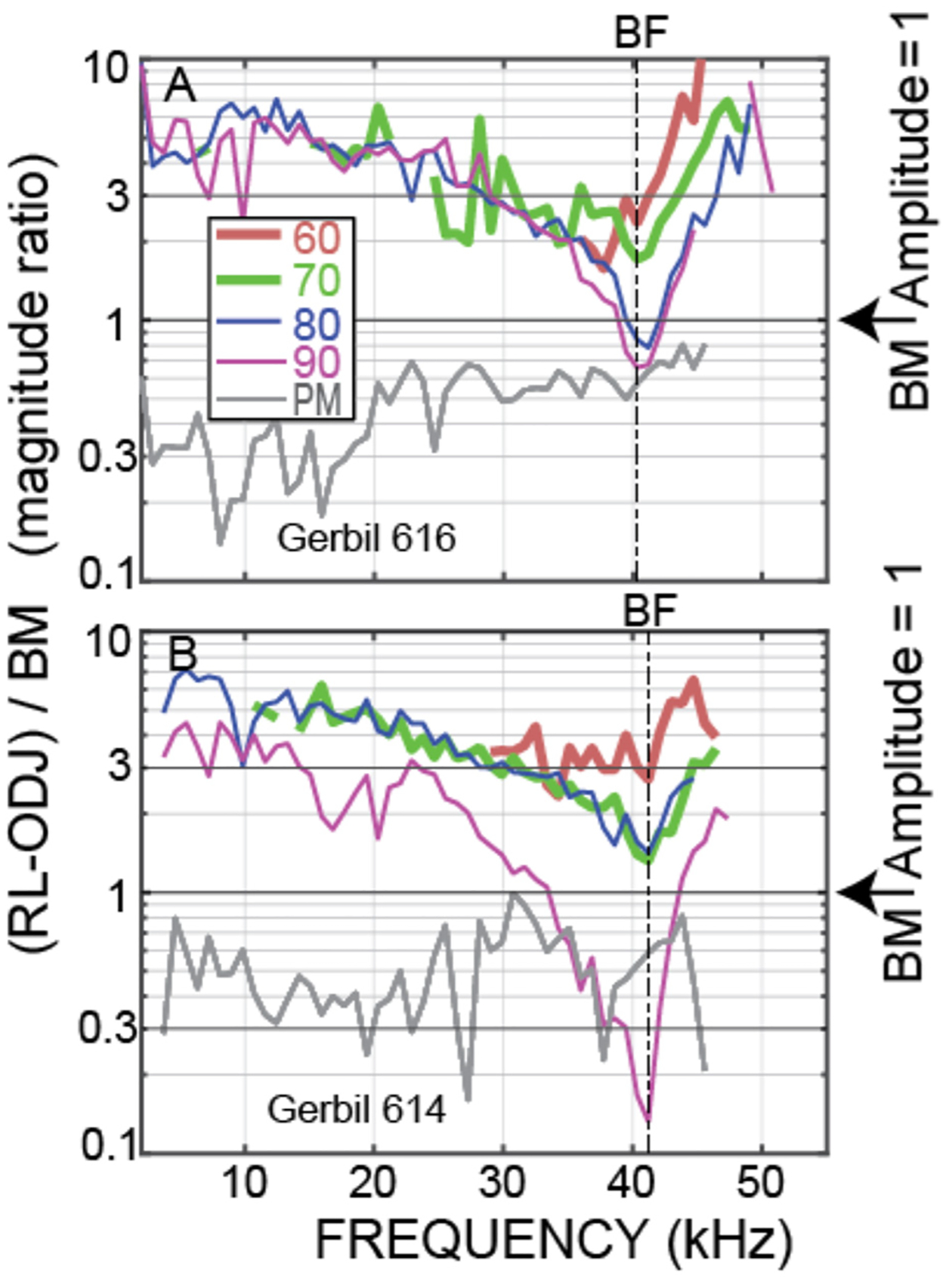
OHC top-to-bottom motion difference relative to BM motion, i.e., (RL-ODJ)/BM, at various sound levels. Data from OHC row 3 of **(A)** Gerbil 616. **(B)** Gerbil 614.

Although the OHC tops and bottoms moved in opposite directions at low frequencies and above BF in the examples of Figure 3, whether this OHC motion has any effect on amplification depends on its amplitude. OHC motion was compared to BM motion using the ratio of the magnitude of OHC top-to-bottom motion, ΔOHC = RL – ODJ, relative to the magnitude of BM motion, i.e., by ΔOHC/BM. Again, by calculating motion ratios, the traveling-wave phases are canceled. Figure 4 shows ΔOHC/BM magnitude for the animals of Figure 3. These data show that, at the lowest sound levels measured, the amplitude of OHC motion exceeded BM motion from the lowest frequencies to above 40 kHz. For the PM condition, OHC motion was less than BM motion for all frequencies measured.

**FIGURE 5:**
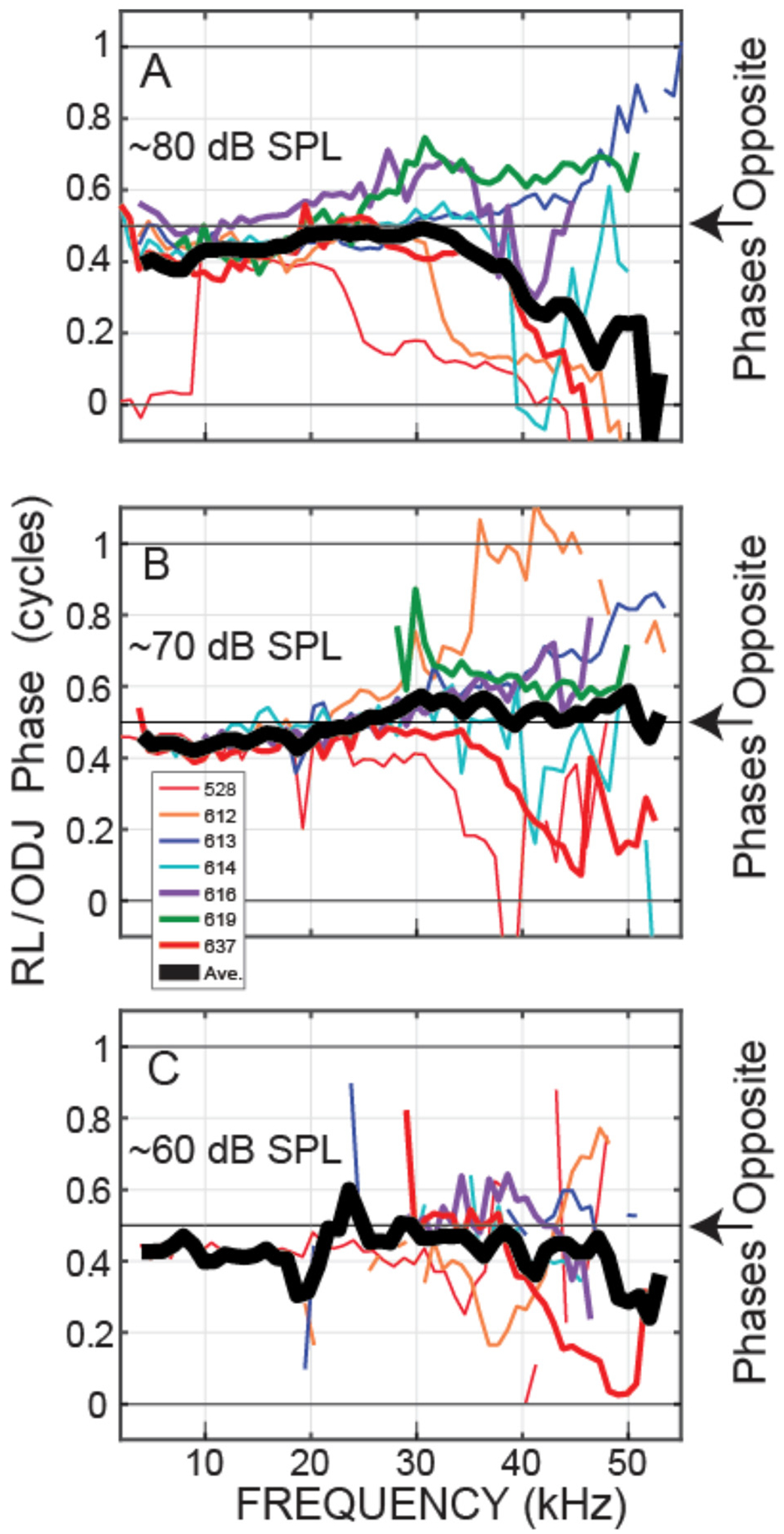
At frequencies up to 45–50 kHz, outer-hair-cell (OHC) top and bottom motions were nearly opposite in phase. The OHC top-to-bottom phase ratios from seven gerbils (animal code in inset). The thick black line is the average (on a linear scale) across the available data in 2 kHz overlapping ranges. Data from nominally 80 **(A)**, 70 **(B)** or 60 **(C)** dB SPL tones.

**FIGURE 6:**
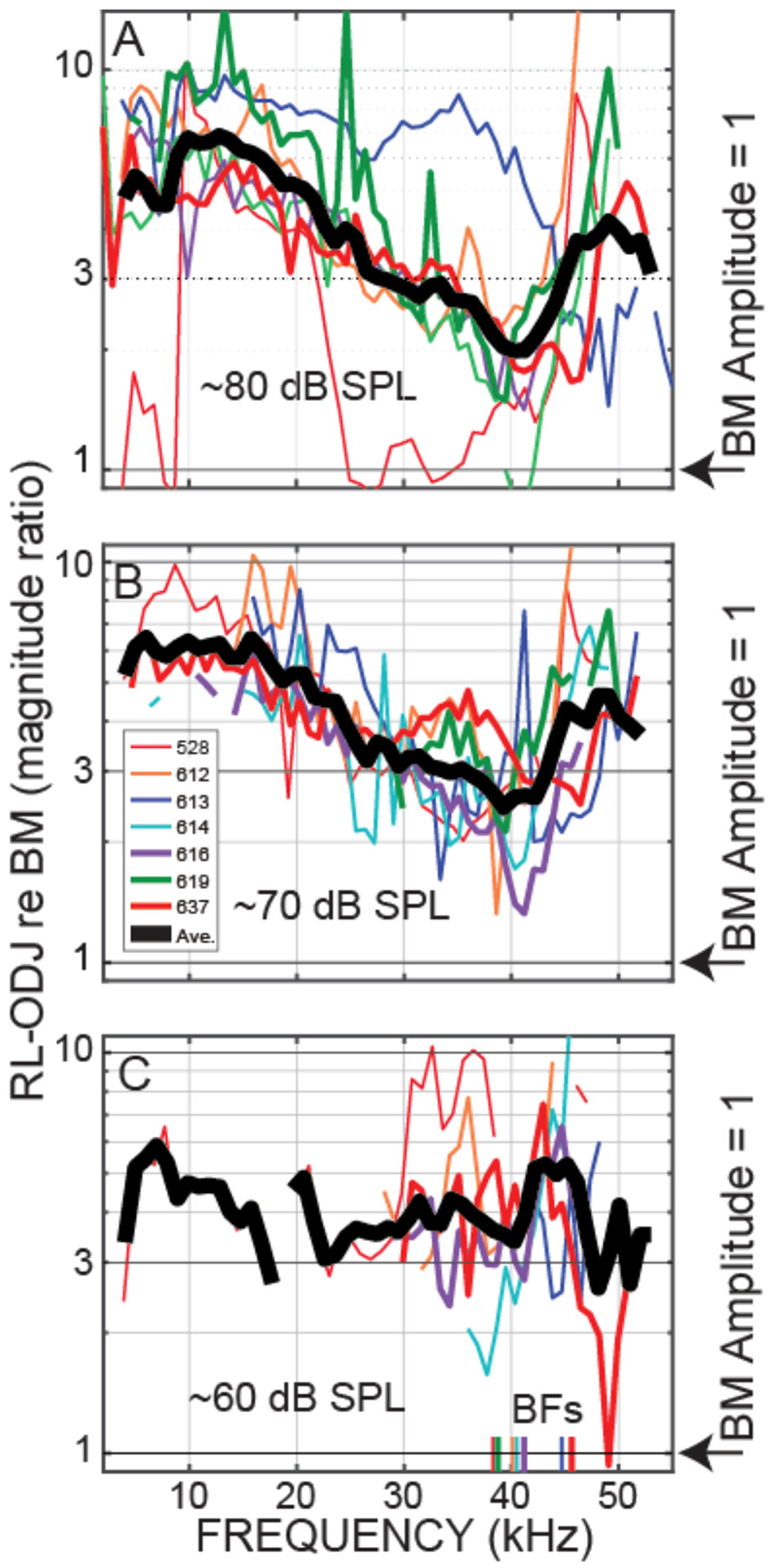
At frequencies up to 45–50 kHz, outer-hair-cell (OHC) top and bottom motion differences were larger than the BM motion. Magnitude ratios of OHC top-to-bottom differential motion re BM motion (ΔOHC/BM). Data from even gerbils (code in inset at top). The thick gray line is the average (on a log scale) across the available data in 2 kHz overlapping ranges. Data from nominally 80 **(A)**, 70 **(B)** or 60 **(C)** dB SPL tones.

Plots at 60, 70, and 80 dB SPL nominal sound levels from all of the animals for which we have these data are shown in Figures 5, 6. Plots of these data as functions of frequency/BF are given in Supplementary material. Figure 5 shows the OHC top-to-bottom phase difference and Figure 6 shows the relative magnitudes of the OHC differential motions to the BM motions. The OHC top-to-bottom phase data (Figure 5) have a lot of scatter at high frequencies, but for 60 and 70 dB SPL, where cochlear amplification has more effect than at 80 dB SPL, the average data (thick black lines) show near a half-cycle difference up to frequencies of 45–50 kHz. Figure 6 shows that throughout the frequency range, in many cases extending to ~50 kHz, the magnitude of the OHC top-to-bottom differential motion was greater than the magnitude of the BM motion. At the 60 dB SPL nominal level, the OHC differential motion, ΔOHC, was typically more than three times greater than the magnitude of the BM motion, across frequencies (Figure 6B). A permutation test of statistical significance (see Section 2) on all available data within the 40–50 kHz range showed average ΔOHC/BM’s for 60, 70, and 80 dB SPL nominal levels of 4.89, 3.82, and 3.38 with the probability that the average ΔOHC/BM would fall below 3 of *P* < 0.0001, *P* < 0.0001, and *P* = 0.087, respectively (i.e., highly significant at 60 and 70 dB and not significant at 80 dB). Using a criterion of 3 is arbitrary, but it serves to illustrate that ΔOHC was substantially greater than BM motion.

## 4 Discussion

We directly measured, *in vivo*, individual OHC top-to-bottom differential motion in the 38–46 kHz region of the gerbil and compared it to BM motion. For moderate-level tones (60 and 70 dB SPL), the differences between the OHC top and bottom motions were close to out-of-phase up to 45–50 kHz (Figures 3, 5), which is consistent with this motion being due to OHC motility and not simply motion that follows BM transverse motion. This OHC motion extended to frequencies more than a decade above the OHC corner frequency in the gerbil cochlear base (Vavakou et al., 2019). Even more important, at moderate sound levels the amplitude of the OHC motion was greater than BM motion, most often by a factor of 3 or more at 60 and 70 dB SPL (Figures 4, 6). Thus, our data show that in response to sound stimulation, single OHCs cyclically expand and contract at substantial amplitudes up to frequencies of 45–50 kHz.

It is interesting to note that at 80 dB SPL, the OHC top-to-bottom phase difference changed from antiphase at low frequencies to near zero at the highest frequencies (Figure 5A). One interpretation of these data is that at high levels near BF, BM motion dominates the OHC differential motion so that the OHC top-to-bottom motion difference is no longer important. This effect can also be seen in the ΔOHC/BM data of Figure 6, which shows that, near BF, the ΔOHC to BM magnitude ratio becomes less as sound level is increased.

We have compared the measured OHC motion to the motion of the BM because OHC-generated forces acting on the BM has been the traditional way to think about cochlear amplification (Robles and Ruggero, 2001; Guinan et al., 2012). However, it has been proposed that cochlear amplification is produced by OHC forces acting to change the local cross-sectional area of the OoC in a way that adds energy to the traveling wave in scala-media/vestibuli (Altoe et al., 2022; Guinan, 2022). With this conception, OHC motion might best be compared to RL motion. Since near BF, RL motion is much greater than BM motion (Figure 2), this would make the OHC top-to-bottom motion be smaller than RL motion. OHC forces could still produce cochlear amplification, e.g. it only takes small pushes each cycle to make a swing move much greater than the movement in the push, Furthermore, cochlear amplification builds up gradually over about ½ octave basal to the BF place, so that at each longitudinal location, only a small increment of gain is added (Yoon et al., 2011; Motallebzadeh et al., 2018). Our measurements don’t tell us how cochlear amplification works, but they do tell us that OHC motion is substantial at 40–50 kHz (Figure 6), and not as sometimes supposed diminished by the OHC RC time constant to be negligibly small.

### 4.1 The OHC-RC-time-constant problem

The so-called “OHC-RC-time-constant problem” was first noted from *in vitro* measurements of OHC somatic motility, and it was assumed that the long RC time constants would limit the effects of OHC force production to just a few kHz (Santos-Sacchi, 1989; Housley and Ashmore, 1992; Vavakou et al., 2019). Alternative arguments were that *in vivo* OHCs are bathed in the fluid environment of the cochlea, and that OHCs act on the internal impedance of the OoC (which is not present in a dish) so that OHC function would be fundamentally different *in vivo* compared to *in vitro* (Mountain and Hubbard, 1994; Iwasa, 2017). *In-vivo* measurements using a zwuis stimulus tone complex spectrum in the gerbil base indicated that the OHCs had a 3 kHz low-pass corner frequency (Vavakou et al., 2019). Since this OHC corner frequency was several octaves lower than the local BF, Vavakou et al. (2019) concluded that OHC motion would not be enough to produce cochlear amplification in high-BF regions (which Figures 5, 6 show is incorrect).

From experiments in gerbils and rats, Johnson et al. (2011) concluded that the OHC RC time constant decreased from apex to base, however they also found that the OHC channel-open probability was near ½ and varied very little across BF regions. An OHC channel-open probability of ½ implies that the resting operating point of the OHC mechano-electric-transfer (MET) function is near the middle of its range, i.e., the MET function is symmetric about its resting operating point. In contrast, in the guinea-pig apex, the OHC MET function was found to be highly asymmetric (Dallos et al., 1982; Dallos, 1985). Furthermore, in live, sensitive cats with unopened cochleae, low-frequency bias-tone effects on auditory-nerve-fiber responses (with bias-tone effects on IHCs excluded) showed a smooth gradation with low-CF regions showing high OHC MET asymmetry and regions with CFs >10–20 kHz showing symmetric OHC MET functions (Cai and Geisler, 1996; Nam and Guinan, 2016). Perhaps the difference in results across the above experiments is due to species (gerbil and rat vs. guinea-pig and cat), but we think it is more likely that the difference is due to an excised-preparation vs. a live-animal preparation. The discrepancy in the symmetry of the OHC MET function between Johnson et al.’s (2011) measurements and those in live animals casts doubt Johnson et al’s other conclusion that the OHC RC time constant varies with CF, or, at least, that it varies as much as Johnson et al proposes. Furthermore, Johnson et al.’s (2011) OHC RC time constants do not agree with the time constant measured by Vavakou et al. (2019) in the 13–25 kHz region of live, sensitive gerbils, where Fc ranged from 2.1 to 3.3 kHz with a weak trend of the RC time constant decreasing with BF. Overall, the extent to which the OHC corner frequency may vary along the length of the cochlea is unknown.

Before to our current work, the highest frequency at which OHC sound-driven motility had been documented was from the mouse 9 kHz region where differential OHC top-to-bottom motions at stimulus harmonics up to 20 kHz were closer to out-of-phase than in-phase (i.e., phase difference >¼ cycle) (Dewey et al., 2021). Our experiments extend the frequency range showing sound-driven OHC motility up to 40–50 kHz, and, most importantly, show that for moderate-level sounds the OHC top-to-bottom differential motion is *larger* than BM motion. Thus, it is feasible that OHC motility drives cycle-by-cycle cochlear amplification even at frequencies far above the “OHC corner frequency” measured in isolated OHCs.

Why is there still substantial OHC motility at frequencies more than an order of magnitude above the OHC corner frequency? One explanation has been that OHCs can work at such high frequencies because the coupling of the load on the OHCs acts to balance out the OHC capacity and increase the effective corner frequency (Mountain and Hubbard, 1994; Iwasa, 2017). When the RC time constant “problem” was identified, the OHC output was considered to be OHC *displacement*, but it has been argued that the OHC output *velocity*, which is the time derivative of displacement and therefore flat above the voltage corner frequency, is what is important for cochlear amplification since OHC velocity would be most closely related to the OHC force at frequencies where there is traveling-wave amplification (Altoè and Shera, 2023). Whether-or-not this is correct, Figure 6 shows that the *displacement* magnitude of the OHC motion is greater than BM *displacement* magnitude up to very high frequencies (and this is also true for velocity). Perhaps the way to think about this issue is that the mechanical energy produced by prestin (which is mechanical energy converted from electrical energy) is not captured by a simple OHC RC time constant because part of the OHC capacitance arises from the mechanism by which electrical energy is converted to mechanical energy (see Rabbitt, 2020, 2022). Whatever the underlying cause, it appears that sound-driven OHCs work well enough to produce substantial motion at frequencies well above their RC time constant.

### 4.2 Comparisons with other data

Ren et al. (2016) reported measurements purported to be from the RL that showed motion with a phase approximately opposite to BM phase at low frequencies. However, their optical system did not provide an image of the live tissue so their assessment that the structure measured was the RL was based on the depth of the reflective structure from the BM compared to the BM to RL depth in histological sections. An interpretation consistent with the data is that Ren et al.’s (2016) so-called “RL” measurements were from the ODJ (i.e. they are like Figure 2B).

Our measurements of OHC motion at high frequencies can be compared to *in vivo* measurements of OHC receptor currents or voltages. In the gerbil ~24 kHz BF region, the extracellular voltage just under the BM (a surrogate for the local OHC current) was measured by Dong and Olson (2013) along with scala-tympani pressure and BM displacement. From these measurements it was surmised that the extracellular voltage and displacements were in phase at low frequencies (more than ½-octave below BF), but at ½-octave below BF there was a rapid phase change so that from ½-octave below BF up to BF, the phase of the local OHC current led BM displacement by ¼ to ½ cycle (Dong and Olson, 2013). Our measurements of the phase of the differential OHC-top-to-bottom motion (Figures 3, 5) do not easily fit with an abrupt transition at ½-octave below BF. The phases in these two experiments seem likely to be related, but we don’t understand exactly how. The two sets of data agree on there being a consistent response phase across frequency at more than an octave below BF.

It has recently been argued that at frequencies above the OHC corner frequency, OHC motility is activated by OHC *extracellular a.c. voltages* that are larger than the OHC intracellular a.c. receptor potential (Levic et al., 2022; Santos-Sacchi et al., 2023). However, the (Levic et al., 2022) report that at frequencies of a few tens of kHz, the extracellular potential was almost an order of magnitude larger than the intra-OHC receptor potential, is difficult to reconcile with the OHC RC filter being intact. At frequencies far above the OHC RC filter corner frequency, the low impedance of the OHC RC filter will reduce the OHC transmembrane voltage no matter whether the electrical source is inside or outside of the OHC. Also, Levic et al.’s (2022) report of almost no OHC transmembrane a.c. voltage difference at low frequencies, doesn’t fit with there being large measured OHC top-to-bottom motion at low frequencies (e.g. Figures 2, 4, 6). How, Levic et al.’s (2022) data fits with these other data is unclear, but we note that the possibility that the electrode penetrating the OHC might have damaged the OHC was never considered in Levic et al. (2022).

## 5 Conclusion

Our measurements show that, *in vivo, individual* OHCs cyclically expand and contract with amplitudes greater than BM motion from 2 kHz up to ~50 kHz. Thus, OHCs are not prevented from amplifying at high frequencies by a low OHC corner frequency.

## Supplementary Material

Data Sheet 1.pdf

The Supplementary Material for this article can be found online at: https://www.frontiersin.org/articles/10.3389/fauot.2025.1617134/full#supplementary-material

## Data Availability

The data for this study will be made available on the Harvard Dataverse site https://doi.org/10.7910/DVN/C0VCPN, or from the corresponding author on request.

## References

[R1] Altoe A, Dewey JB, Charaziak KK, Oghalai JS, and Shera CA (2022). Overturning the mechanisms of cochlear amplification via area deformations of the organ of Corti. J. Acoust. Soc. Am 152:2227. doi: 10.1121/10.0014794 36319240 PMC9578757

[R2] Altoè A, and Shera CA (2023). The long outer-hair-cell rc time constant: a feature, not a bug, of the mammalian cochlea. J. Assoc. Res. Otolaryngol 24, 129–145. doi: 10.1007/s10162-022-00884-w 36725778 PMC10121995

[R3] Brownell WE, Bader CR, Bertrand D, and de Ribaupierre Y (1985). Evoked mechanical response of isolated cochlear outer hair cells. Science 277, 194–196. doi: 10.1126/science.3966153 3966153

[R4] Cai Y, and Geisler CD (1996). Suppression in auditory-nerve fibers of cats using low-side suppressors. I. Temporal aspects. Hear. Res 96, 94–112. doi: 10.1016/0378-5955(96)00034-2 8817310

[R5] Cho NH, and Puria S (2022). Cochlear motion across the reticular lamina implies that it is not a stiff plate. Sci. Rep 12:18715. doi: 10.1038/s41598-022-23525-x 36333415 PMC9636238

[R6] Cho NH, Wang H, and Puria S (2022). Cochlear fluid spaces and structures of the gerbil high-frequency region measured using optical coherence tomography (OCT). J. Assoc. Res. Otolaryngol 23, 195–211. doi: 10.1007/s10162-022-00836-4 35194695 PMC8964889

[R7] Cooper NP, Vavakou A, and van der Heijden M (2018). Vibration hotspots reveal longitudinal funneling of sound-evoked motion in the mammalian cochlea. Nat. Commun 9:3054. doi: 10.1038/s41467-018-05483-z 30076297 PMC6076242

[R8] Dallos P (1985). Response characteristics of mammalian cochlear hair cells. J. Neurosci 5, 1591–1608. doi: 10.1523/JNEUROSCI.05-06-01591.1985 4009248 PMC6565270

[R9] Dallos P, Santos-Sacchi J, and Flock Å (1982). Intracellular recordings from cochlear outer hair cells. Science 218, 582–584. doi: 10.1126/science.7123260 7123260

[R10] Dewey JB, Altoè Altoè A, Shera CA, Applegate BE, and Oghalai JS (2021). Cochlear outer hair cell electromotility enhances organ of corti motion on a cycle-by-cycle basis at high frequencies *in vivo*. Proc. Natl. Acad. Sci. USA 118:e2025206118. doi: 10.1073/pnas.2025206118 34686590 PMC8639341

[R11] Dong W, and Olson ES (2013). Detection of cochlear amplification and its activation. Biophys. J 105, 1067–1078. doi: 10.1016/j.bpj.2013.06.049 23972858 PMC3752116

[R12] Efron B, and Tibshirani RJ (1993). An Introduction to the Bootstrap. New York, NY: Chapman and Hall. doi: 10.1007/978-1-4899-4541-9

[R13] Frank G, Hemmert W, and Gummer AW (1999). Limiting dynamics of high-frequency electromechanical transduction of outer hair cells. Proc. Natl. Acad. Sci. USA 96, 4420–4425. doi: 10.1073/pnas.96.8.4420 10200277 PMC16347

[R14] Gale JE, and Ashmore JF (1994). Charge displacement induced by rapid stretch in the basolateral membrane of the guinea-pig outer hair cell. Proc. R Soc. London B 255, 243–249. doi: 10.1098/rspb.1994.0035 8022840

[R15] Guinan JJ Jr. (2022). Cochlear amplification in the short-wave region by outer hair cells changing organ-of-corti area to amplify the fluid traveling wave. Hear. Res 426:108641. doi: 10.1016/j.heares.2022.108641 39776694 PMC11706524

[R16] Guinan JJ Jr., Cho NH, and Puria S (2025). The reduced cortilymph flow path in the short-wave region allows outer hair cells to produce focused traveling-wave amplification. J. Assoc. Res. Otolaryngol 26, 49–61. doi: 10.1007/s10162-025-00976-3 39920422 PMC11861466

[R17] Guinan JJ Jr., Salt A, and Cheatham MA (2012). Progress in cochlear physiology after Bekesy. Hear. Res 293, 12–20. doi: 10.1016/j.heares.2012.05.005 22633944 PMC3530189

[R18] Housley GD, and Ashmore JF (1992). Ionic currents of outer hair cells isolated from the guinea pig cochlea. J. Physiol 448, 73–98. doi: 10.1113/jphysiol.1992.sp019030 1593487 PMC1176188

[R19] Iwasa KH (2017). Negative membrane capacitance of outer hair cells: electromechanical coupling near resonance. Sci. Rep 7:12118. doi: 10.1038/s41598-017-12411-6 28935970 PMC5608895

[R20] Johnson SL, Beurg M, Marcotti W, and Fettiplace R (2011). Prestin-driven cochlear amplification is not limited by the outer hair cell membrane time constant. Neuron 70, 1143–1154. doi: 10.1016/j.neuron.2011.04.024 21689600 PMC3143834

[R21] Levic S, Lukashkina VA, Simoes P, Lukashkin AN, and Russell IJ (2022). A gap-junction mutation reveals that outer hair cell extracellular receptor potentials drive high-frequency cochlear amplification. J. Neurosci 42, 7875–7884. doi: 10.1523/JNEUROSCI.2241-21.2022 36261265 PMC9617611

[R22] Motallebzadeh H, Soons JAM, and Puria S (2018). Cochlear amplification and tuning depend on the cellular arrangement within the organ of Corti. Proc. Natl. Acad. Sci. USA 115, 5762–5767. doi: 10.1073/pnas.1720979115 29760098 PMC5984506

[R23] Mountain DC, and Hubbard AE (1994). A piezoelectric model of outer hair cell function. J. Acoust. Soc. Am 95, 350–354. doi: 10.1121/1.408273 8120246

[R24] Nam H, and Guinan JJ Jr. (2016). Low-frequency bias tone suppression of auditory-nerve responses to low-level clicks and tones. Hear. Res 341, 66–78. doi: 10.1016/j.heares.2016.08.007 27550413 PMC5086432

[R25] Rabbitt RD (2020). The cochlear outer hair cell speed paradox. Proc. Natl. Acad. Sci. USA 117, 21880–21888. doi: 10.1073/pnas.2003838117 32848062 PMC7486750

[R26] Rabbitt RD (2022). Analysis of outer hair cell electromechanics reveals power delivery at the upper-frequency limits of hearing. J. R. Soc. Interface 19:20220139. doi: 10.1098/rsif.2022.0139 35673856 PMC9174718

[R27] Ren T, He W, and Kemp D (2016). Reticular lamina and basilar membrane vibrations in living mouse cochleae. Proc. Natl. Acad. Sci. USA 113, 9910–9915. doi: 10.1073/pnas.1607428113 27516544 PMC5024575

[R28] Robles L, and Ruggero MA (2001). Mechanics of the mammalian cochlea. Physiol. Rev 81, 1305–1352. doi: 10.1152/physrev.2001.81.3.1305 11427697 PMC3590856

[R29] Santos-Sacchi J (1989). Asymmetry in voltage dependent movements of isolated outer hair cells from the organ of Corti. J. Neurosci 9, 2954–2962. doi: 10.1523/JNEUROSCI.09-08-02954.1989 2769373 PMC6569687

[R30] Santos-Sacchi J (1992). On the frequency limit and phase of outer hair cell motility: effects of the membrane filter. J. Neurosci 12, 1906–1916. doi: 10.1523/JNEUROSCI.12-05-01906.1992 1578277 PMC6575887

[R31] Santos-Sacchi J, Bai JP, and Navaratnam D (2023). Megahertz sampling of prestin (SLC26a5) voltage-sensor charge movements in outer hair cell membranes reveals ultrasonic activity that may support electromotility and cochlear amplification. J. Neurosci 43, 2460–2468. doi: 10.1523/JNEUROSCI.2033-22.2023 36868859 PMC10082455

[R32] Santos-Sacchi J, and Tan W (2018). The frequency response of outer hair cell voltage-dependent motility is limited by kinetics of prestin. J. Neurosci 38, 5495–5506. doi: 10.1523/JNEUROSCI.0425-18.2018 29899032 PMC6001036

[R33] Santos-Sacchi J, and Tan W (2019). Voltage does not drive prestin (SLC26a5) electro-mechanical activity at high frequencies where cochlear amplification is best. iScience 22, 392–399. doi: 10.1016/j.isci.2019.11.036 31812809 PMC6911985

[R34] van der Heijden M, and Vavakou A (2021). Rectifying and sluggish: outer hair cells as regulators rather than amplifiers. Hear. Res 2:108367. doi: 10.1016/j.heares.2021.108367 34686384

[R35] Vavakou A, Cooper NP, and van der Heijden M (2019). The frequency limit of outer hair cell motility measured *in vivo*. Elife. 8:e023. doi: 10.7554/eLife.47667.023 PMC6759357 31547906

[R36] Wang Y, Steele CR, and Puria S (2016). Cochlear outer-hair-cell power generation and viscous fluid loss. Sci. Rep 6:19475. doi: 10.1038/srep19475 26792556 PMC4726291

[R37] Yoon YJ, Steele CR, and Puria S (2011). Feed-forward and feed-backward amplification model from cochlear cytoarchitecture: an interspecies comparison. Biophys. J 100, 1–10. doi: 10.1016/j.bpj.2010.11.039 21190651 PMC3010833

