# Supplementary material for: In vivo evidence of outer hair cell length changes and their role in high-frequency cochlear mechanics
Source: Front Audiol Otol. Author manuscript; Available in PMC 2026 May 29. (PMC13218775; doi:10.3389/fauot.2025.1617134)
Supplement: Data Sheet 1.pdf [file NIHMS2177806-supplement-Data_Sheet_1_pdf.pdf]

Supplementary Material for:

In Vivo Evidence of Outer Hair Cell Length Changes and their Role in High-Frequency Cochlear Mechanics

By
Sunil Puria, Nam Hyun Cho and John Guinan

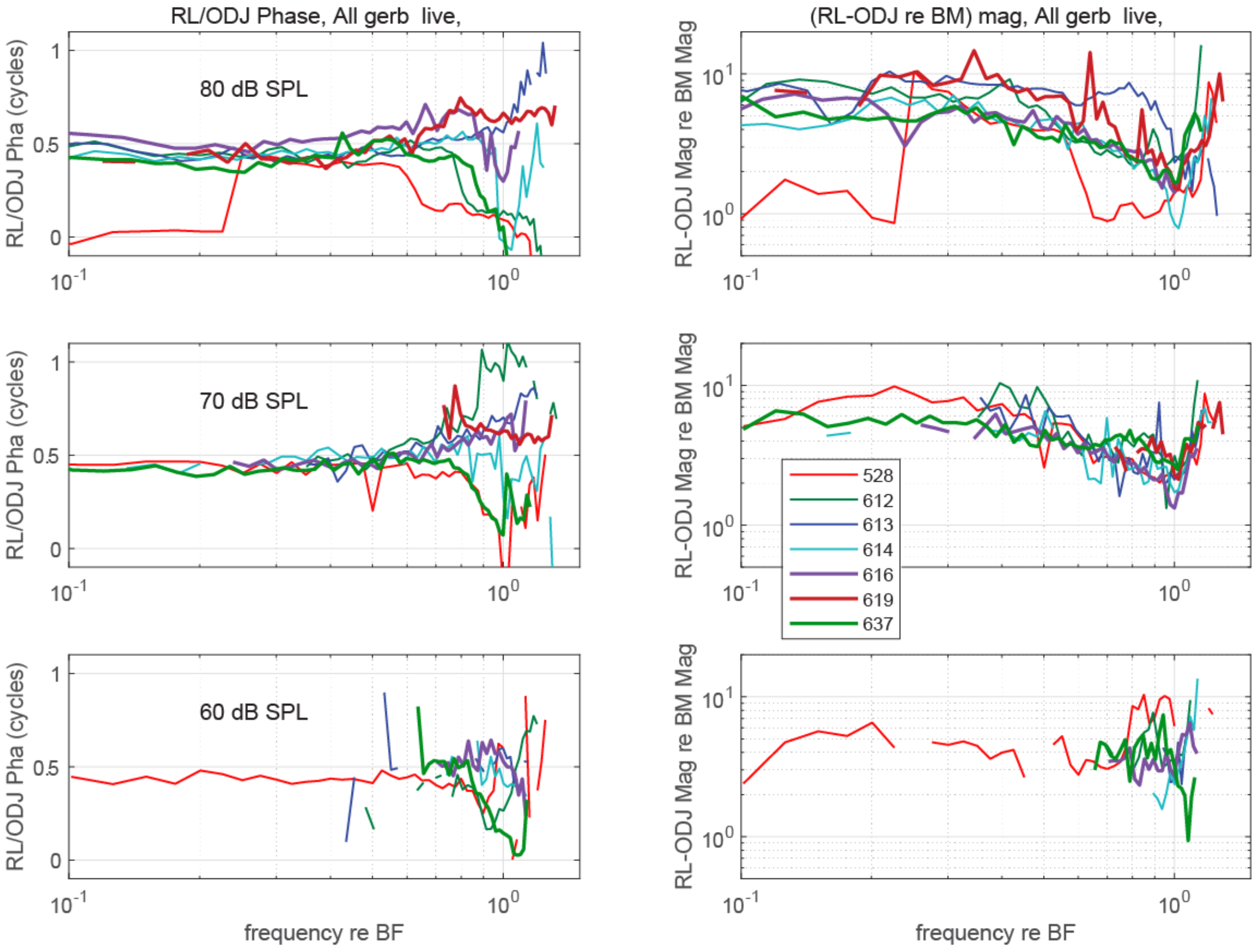

Figure SM1. LEFT: The OHC top-to-bottom phase ratios from 7 gerbils (animal code in inset at right). RIGHT: Magnitude ratios of OHC top-to-bottom differential motion re BM motion (DOHC / BM). Data from 7 gerbils (code in inset). Data from nominally 80 (A), 70 (B) or 60 (C) dB SPL tones normalized to best frequency (BF).
